# Automated solid‐phase synthesis of metabolically stabilized triazolo‐peptidomimetics

**DOI:** 10.1002/psc.3488

**Published:** 2023-03-29

**Authors:** Xabier Guarrochena, Barbara Kaudela, Thomas L. Mindt

**Affiliations:** ^1^ Department of Inorganic Chemistry, Faculty of Chemistry University of Vienna Vienna Austria; ^2^ Vienna Doctoral School in Chemistry University of Vienna Vienna Austria; ^3^ Ludwig Boltzmann Institute Applied Diagnostics AKH Wien c/o Sekretariat Nuklearmedizin Vienna Austria; ^4^ Department of Biomedical Imaging and Image Guided Therapy, Division of Nuclear Medicine Medical University of Vienna Vienna Austria; ^5^ Joint Applied Medicinal Radiochemistry Facility University of Vienna, Medical University of Vienna Vienna Austria

**Keywords:** automated solid‐phase peptide synthesis, CuAAC, diazo‐transfer, triazolo‐peptidomimetics

## Abstract

The use of 1,4‐disubstituted 1,2,3‐triazoles as *trans*‐amide bond surrogates has become an important tool for the synthesis of metabolically stabilized peptidomimetics. These heterocyclic bioisosters are generally incorporated into the peptide backbone by applying a diazo‐transfer reaction followed by CuAAC (click chemistry) with an α‐amino alkyne. Even though the manual synthesis of backbone‐modified triazolo‐peptidomimetics has been reported by us and others, no procedure has yet been described for an automated synthesis using peptide synthesizers. In order to efficiently adapt these reactions to an automated setup, different conditions were explored, putting special emphasis on the required long‐term stability of both the diazo‐transfer reagent and the Cu(I) catalyst in solution. ISA·HCl is the reagent of choice to accomplish the diazo‐transfer reaction; however, it was found instable in DMF, the most commonly used solvent for SPPS. Thus, an aqueous solution of ISA·HCl was used to prevent its degradation over time, and the composition in the final diazo‐transfer reaction was adjusted to preserve suitable swelling conditions of the resins applied. The CuAAC reaction was performed without difficulties using [Cu (CH_3_CN)_4_]PF_6_ as a catalyst and TBTA as a stabilizer to prevent oxidation to Cu(II). The optimized automated two‐step procedure was applied to the synthesis of structurally diverse triazolo‐peptidomimetics to demonstrate the versatility of the developed methodology. Under the optimized conditions, five triazolo‐peptidomimetics (8–5 amino acid residues) were synthesized efficiently using two different resins. Analysis of the crude products by HPLC‐MS revealed moderate to good purities of the desired triazolo‐peptidomimetics (70–85%). The synthesis time ranged between 9 and 12.5 h.

## Graphical abstract

An optimized automated two‐step procedure for the synthesis of structurally diverse triazolo‐peptidomimetics was developed. Under the optimized conditions, five triazolo‐peptidomimetics with 8–5 amino acid residues were synthesized efficiently using two different resins.

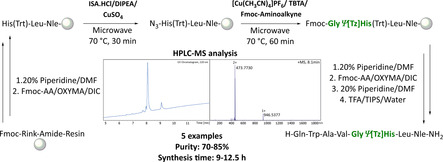

**Abbreviations**
AA: amino acid
ACN: acetonitrile
Bn: benzyl
Boc: tert.butyloxycarbonyl
CuAAC: Copper(I) catalyzed azide‐alkyne cycloaddition
DCC: N,N′‐dicyclohexylcarbodiimide
DCM: dichloromethane
DIC: diisopropylcarbodiimide
DIPEA: N,N‐diisopropylethylamine
DMAP: 4‐dimethylamino pyridine
DMF: *N*,*N*‐dimethylformamide
DMSO: dimethyl sulfoxide
FA: formic acid
Fmoc: 9‐fluorenylmethyloxycarbonyl
h: hour
HOBt: 1‐hydroxybenzotriazole
HPLC: high pressure liquid chromatography
ISA.HCl: imidazole‐1‐sulfonyl azide hydrochloride
LC‐MS: liquid chromatography‐mass spectrometry
MBHA: methylbenzhydrylamine
MeOH: methanol
min: minutes
MS: mass spectrometry
MTBE: methyl‐tert.butyl ether
MW: microwave
OXYMA: ethyl cyanohydroxyiminoacetate
Pbf: 2,2,4,6,7‐pentamethyldihydrobenzofuran‐5‐sulfonyl
PEG: polyethylene glycole
PG: protecting group
PS: polystyrene
r_t_
: retention time
RT: room temperature
SPPS: solid‐phase peptide synthesis
TBTA: tris[(1‐benzyl‐1*H*‐1,2,3‐triazol‐4‐yl)methyl]amine
tBu: tert.butyl
TFA: trifluoroacetic acid
TIPS: triisopropylsilane
Trt: trityl
Tz: triazole

1

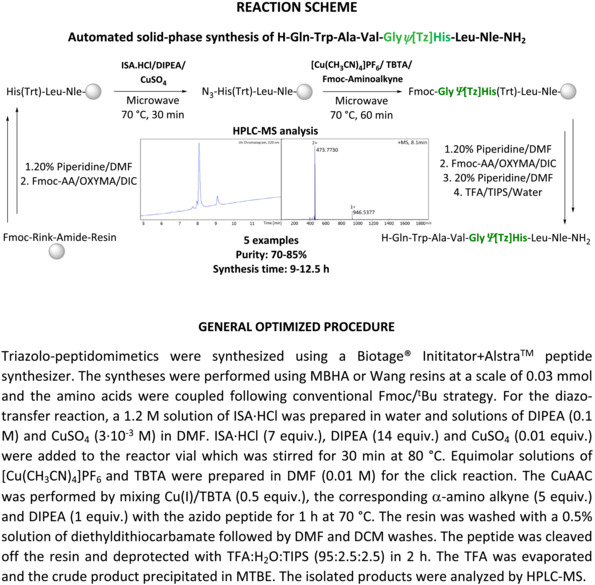

## 2 SCOPE AND COMMENTS

**FIGURE 1:**
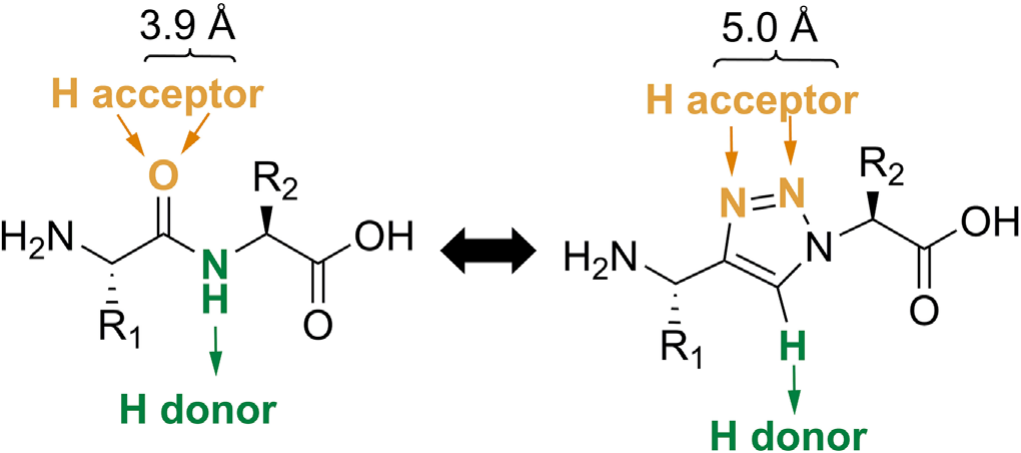
Comparison between the *trans*‐amide bond (left) and the 1,4‐disubstituted 1,2,3‐triazole (right). The orange arrows indicate H‐bond acceptors, and the green arrows the H‐bond donors. The length of the functional groups is specified in Angstroms (Å). R_1_ and R_2_ correspond to different (protected) side chains specific for an amino acid.

The importance of peptides in medicine has notably grown.^1^ Their ability to interact with clinically relevant proteins with exquisite affinity and specificity make them ideal drug candidates. Peptides are also excellent vehicles (vectors) to transport selectively imaging probes and cytotoxic payloads to the site of diseases due to their suitable pharmacokinetic profiles and tissue penetration properties, as well as convenient and economic availability.^2^ However, the use of peptides in the clinic is hampered by their metabolic lability, which can limit their accumulation at the biological target(s).^3^ As a result, many research groups have reported different methods for the stabilization of peptides, for example, by structural modifications (e.g., N‐methylation^4^ or reduction of amide bonds, employment of amide bond surrogates, or incorporation of unnatural amino acids^5^) and/or conformational restraints (e.g., head‐to‐tail cyclization and stapling^6^). The use of metabolically stable amide bond bioisosters represents an attractive approach to enhance the metabolic stability of a peptide while preserving its biological function(s). Among the examples reported (e.g., sulfonamides and semicarbazides^7^), 1,4‐disubstituted 1,2,3‐triazoles (Tz) have been shown to be suitable *trans*‐amide bond surrogates.^8^ On the other hand, 1,5‐disubstituted 1,2,3‐trizoles can serve as analogs of *cis*‐amine bonds.^9^ These metabolically stable Tz‐based bioisosters of amide bonds share similarities with the amido functional group in terms of size, planarity, H‐bonding properties, and polarity (Figure 1).

The incorporation of triazoles into the peptide's backbone can be accomplished in different ways. An elegant approach relies on the use of solid‐phase chemistry for both peptide elongation and triazole incorporation. This way, the peptide is elongated following standard Fmoc/^t^Bu chemistry until the position of Tz‐insertion, and this is accomplished on solid support in two sequential synthetic steps. The first reaction makes use of a diazo‐transfer reagent (e.g., imidazole‐1‐sulfonyl azide^10^) for the amide‐to‐azide conversion at the N‐terminus of the peptide. The second reaction consists of the [2+3] dipolar cycloaddition between the introduced azide functionality and an appropriate alkyne derivatives of amino acids.^11^ The in solution synthesis of α‐amino alkynes starting from commercial amino acid derivatives is described elsewhere.^12^ Employment of specific metal catalysts yields different regioisomers of the triazole heterocycle. While Copper(I)^13^ is used for the synthesis of 1,4‐disubstituted 1,2,3‐triazoles, Ruthenium(II)^14^ has proven useful to synthesize the 1,5‐disubstituted regioisomers. Albeit reported,^15^ the introduction of 1,5‐disubstituted 1,2,3‐triazoles into peptides on solid‐phase is challenging, at least in our hands.^16^ We therefore focus here on the application of 1,4‐disubstituted 1,2,3‐triazoles as *trans*‐amide bond surrogates.

The emergence and widespread availability of automated peptide synthesizers offers an opportunity for the rapid synthesis of not only peptides but also peptidomimetics, provided that the chemistry used is compatible with SPPS. Even though the diazo‐transfer^17^ reaction and CuAAC^18^ have been reported for the synthesis of triazolo‐peptidomimetics by manual SPPS, they have not yet been adapted to an automated setup. Here, we report a robust protocol for the fast and facile automated solid‐phase synthesis of backbone modified triazole‐bearing peptidomimetics.

**FIGURE 2:**
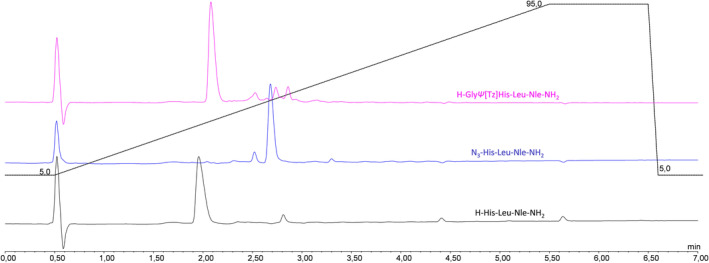
UV (220 nm)/HPLC chromatogram of H_2_N‐His‐Leu‐Nle‐NH_2_ (r_t_ = 1.96 min), N_3_‐His‐Leu‐Nle‐NH_2_ (r_t_ = 2.68 min), and H_2_N‐Gly*Ψ*[Tz]His‐Leu‐Nle‐NH_2_ (r_t_ = 2.08 min).

The compatibility of the diazo‐transfer and CuAAC reactions with an automated synthesis setup was studied with the model peptide sequence H‐Gly*Ψ*[Tz]His‐Leu‐Nle‐NH_2._ ^19^ Unlike in manual synthesis, all solutions of reagents need to be prepared in advance, and the long‐term stability of the reagents is essential for automated and programmed reaction sequences. The first attempts to perform the diazo‐transfer reaction were conducted in DMF using ISA·HCl (5 equiv.) and DIPEA (10 equiv.) with N‐terminally deprotected H‐His (Trt)‐Leu‐Nle‐NH_2_ bound to a MBHA resin. These conditions did not yield the desired triazolo‐peptidomimetic, and an insufficient stability of the diazo‐transfer reagent was suspected to be the limiting factor. Subsequent stability studies of ISA·HCl in DMF (HPLC‐grade) revealed a significant degradation of the sulfonyl azide to the corresponding sulfonyl amine (HPLC‐MS analysis). Only 30% of the diazo‐transfer reagent remained intact after 4 h of incubation in DMF at RT (22°C) (Figure S1). Thus, the storage of ISA·HCl solutions in the peptide synthesizer for the insertion of Tz in the N‐terminal region of medium or longer length peptides turned out not to be an option. Similarly, DMSO was not able to preserve the integrity of ISA·HCl over time, and thus, these alternative solvents for SPPS were not followed up further. In contrast, ISA·HCl was found sufficiently stable in aqueous solutions with >80% of intact sulfonyl azide remaining after 24 h at RT in the presence of oxygen (air). In order to counteract potential issues with the swelling properties of polystyrene resins in the presence of water, the water content in the final reaction mixture was minimized to achieve a DMF‐to‐water ratio of 91:9 (total 3 ml DMF from solutions of DIPEA and 300 μl H_2_O from the ISA·HCl solution). With the aqueous solution of ISA·HCl and applying the same reaction conditions as described above, the conversion of the N‐terminal amine of the peptide to the azido peptide (N_3_‐His‐Leu‐Nle‐NH_2_) was 95%, whereas the crude purity determined by HPLC‐MS was >85%. The reaction conditions were further optimized by screening the use of different equiv. of employed ISA·HCl and DIPEA, varying the temperature and time, as well as the utilization of CuSO_4_ catalyst.^20^ Under the optimized conditions as shown in Table 1, approximately 99% conversion of the N‐terminal amine of the peptide to an azido‐functionality was achieved providing the azido‐peptide in 90% purity (Figure 2; Table 1, entry 5).

**TABLE 1:** Different reaction conditions examined for the diazo‐transfer reaction.

Entry	ISA·HCl [equiv.]	DIPEA [equiv.]	Reaction time	Temperature (MW)	CuSO_4_ [equiv.]	Conversion	Crude^a^ purity
1	5	10	60 min	RT	‐	95%	85%
2	7	14	60 min	RT	‐	95%	85%
3	7	14	30 min	70°C	‐	68%	61%
4	7	14	30 min	80°C	‐	68%	70%
5	7	14	30 min	80°C	0.01	99%	90%

*Note*: The most efficient conditions are highlighted in green color.

^a^ The crude purity was determined by HPLC‐MS.

Next, we turned our attention to the optimization of the CuAAC on solid support adopted to an automated setup. Similar to the case of the diazo‐transfer reagent, the stability of the Cu(I) catalyst in solution is crucial. [Cu(CH_3_CN)_4_]PF_6_ was chosen as Cu(I) source due to its commercial availability and reported suitable shelf‐life. However, the weakly bound acetonitrile ligands easily dissociate in solution exposing the metal center and therefore posing the risk of the formation of unreactive Cu(II) species. In order to avoid deactivation of the Cu(I) catalysts by oxidation to Cu(II) upon exposure to air, TBTA^21^ was used as stabilizing ligand. TBTA forms a stable complex with Cu(I) and preserves the oxidation state of the metal while making it accessible for catalysis. The automated CuAAC between the azido group at the N‐terminus of the peptide N_3_‐His‐Leu‐Nle‐NH_2_ on solid support and the corresponding α‐amino alkyne were done with a pre‐prepared equimolar solution of [Cu(CH_3_CN)_4_]PF_6_ and TBTA in DMF (0.01 M). Automated addition of solutions of the catalyst, the α‐amino alkyne (Table 2, entry 1: Fmoc‐protected propargyl amine as Gly analog), and DIPEA, the reaction was allowed to proceed for 1 h at 70°C. The triazolo‐peptidomimetic H‐Gly*Ψ*[Tz]His‐Leu‐Nle‐NH_2_ was obtained with a conversion of 82% and a purity of 85%. All attempts to shorten the reaction time by varying the temperature and/or the amount of reagents/catalyst did not result in an improvement (Table 2, entries 2–4). Therefore, the initially tested conditions (Table 2, entry 1) were applied for all further experiments. Only in the case of storage of the solution containing the catalyst [Cu(I)(TBTA)] for more than 24 h, a slight decrease of the efficiency of the CuAAC was observed (76% of crude purity; Figure S2). Therefore, no special precautions had to be applied for the automated CuAAC on solid support.

**TABLE 2:** Reaction conditions tested during the CuAAC reaction.

Entry	[Cu(CH_3_CN)_4_]PF_6_ [equiv.]	TBTA [equiv.]	DIPEA [equiv.]	Amino alkyne [equiv.]	Reaction time	Temperature (MW)	Conversion	Crude^a^ purity
1	0.5	0.5	1	5	60 min	70°C	97%	83%
2	0.5	0.5	1	5	30 min	70°C	71%	72%
3	1.5	1.5	1	5	30 min	70°C	72%	73%
4	1	1	1	5	30 min	80°C	72%	79%

*Note*: The most efficient one is highlighted in green color.

^a^ The crude purity was determined by HPLC‐MS.

The optimized two‐step automated procedure was applied to the synthesis of different triazolo‐peptidomimetics in order to demonstrate the general applicability of our new methodology. First, we looked at the application of different resins. Thus, the triazole‐containing minimal binding sequence of bombesin^19^ was synthesized on MBHA and Wang resins (Table 3, entries 1 and 2). In both cases, the desired triazolo‐peptidomimetic was obtained in a total synthesis time of 10–11 h with comparable purities. Thus, no preference for either resin became apparent. In the following, the procedure was also applied to the automated solid‐phase synthesis of reported triazole‐containing analogs of Leu‐enkephalin^22^ and neurotensin^23^ (Table 3, entries 3–4). Together with the above discussed bombesin derivate, the chosen examples represent the application of different α‐amino alkynes without and with side chain functionalities as well as of varying steric demands. At the same time, we varied the position of the triazole in the amino acid sequence relative to the resin to investigate the impact of potential steric effects on the yields and to confirm the stability of reagents in solution when stored for different time periods in the peptide synthesizer. Finally, we implemented the methodology for the synthesis of a minigastrin derivative incorporating multiple triazoles (Table 3, entry 5).^24^, ^25^ In all cases, the desired triazolo‐peptidomimetics were obtained within reasonable time (max. overnight), with good conversion for both standard SPPS (Fmoc/^t^Bu chemistry) and the diazo‐transfer/CuAAC steps (S3–S7).

**TABLE 3:** Summary of the results for the synthesized triazolo‐peptidomimetics.

Entry	Triazolo‐peptidomimetic	Resin	α‐Amino alkyne	Synthesis time (h)	Crude purity^a^ (%)
1	H‐Gln^1^‐Trp^2^‐Ala^3^‐Val^4^‐Gly^5^ *Ψ*[Trz]His^6^‐Leu^7^‐Nle^8^‐NH_2_	MBHA	Gly	10.0	85
2	H‐Gln^1^‐Trp^2^‐Ala^3^‐Val^4^‐Gly^5^ *Ψ*[Trz]His^6^‐Leu^7^‐Nle^8^‐OH	Wang	Gly	11.0	80
3	H‐Tyr^1^‐Gly^2^‐Gly^3^‐Phe^4^ *Ψ*[Trz]Leu^5^‐OH	Wang	Phe	8.5	90
4^b^	H‐Arg^1^ *Ψ*[Trz]Arg^2^‐Pro^3^‐Tyr^4^‐Ile^5^‐Leu^6^‐NH_2_	MBHA	Arg	9.0	70
5	H‐Glu^1^ *Ψ*[Trz]Ala^2^‐Tyr^3^ *Ψ*[Trz]Gly^4^‐Trp^5^‐Nle^6^‐Gln^7^‐Phe^8^‐NH_2_	MBHA	Glu, Tyr	12.5	79

Abbreviations: Gly, Fmoc‐Gly‐CCH; Phe, Fmoc‐Phe‐CCH; Arg, Fmoc‐Arg (Boc)_2_‐CCH; Glu, Fmoc‐Glu (^t^Bu)‐CCH; Tyr, Fmoc‐Tyr (^t^Bu)‐CCH.

^a^ Crude purity of the final triazolo‐peptidomimetic after overall deprotection and cleavage from the resin was determined by HPLC‐MS.

^b^ Given the limited stability of Fmoc‐Arg (Boc)_2_‐CCH in DMF, the amino alkyne solution was prepared shortly before the CuAAC reaction.

In conclusion, we have developed a robust and versatile protocol for the automated synthesis of triazolo‐peptidomimetics on solid support using a microwave‐assisted peptide synthesizer. The protocol is applicable to the synthesis of structurally diverse triazolo‐peptidomimetics containing one or more 1,4‐disubstituted 1,2,3‐triazoles in their backbone, and so far, no limitations have been identified. We encourage the peptide chemistry community to apply the new and convenient methodology in the future to get rapid access to this promising class of metabolically stabilized peptidomimetics.

## 3 EXPERIMENTAL PROCEDURE

### 3.1 General procedure for automated synthesis of triazolo‐peptidomimetics

The synthesis of all triazolo‐peptidomimetics was performed automatically with the microwave‐assisted peptide synthesizer Biotage® Initiator + Alstra™ (for an example of a synthesis report example, see the supporting information) by a combination of standard Fmoc/^t^Bu chemistry, diazo‐transfer reaction, and CuAAC. The reactions were carried out in 10 ml vials at a scale of 0.03 mmol. The Rink amide MBHA (4‐methylbenzylhydrylamine) resin (0.065 mmol/g loading, 100–200 mesh), and the Wang resin (0.99 mmol/g loading, 4‐Benzyloxybenzyl alcohol polystyrene, 200–400 mesh) were used as solid supports. The resins were swollen in DMF at 70°C for 20 min. The Wang resin was preloaded using a cocktail consisting of the first amino acid (5 equiv.), DIC (5 equiv.), HOBt (5 equiv.), and DMAP (0.5 equiv.) in 6 ml DMF and heating at 75°C for 20 min. The cleavage of the Fmoc protecting group was accomplished using 2 × 4.5 ml 20% piperidine in DMF at RT for a 3 and 10 min reaction time, respectively. After the Fmoc‐deprotection, the resin was washed with DMF (2 × 4.5 ml). The amino acids (5 equiv.) were prepared in 0.1 M solutions in DMF. OXYMA (0.2 M in DMF) and DIC (0.05–2 M in DMF) were used as coupling reagents, and five equivalents of each were used in each reaction. Coupling reactions were carried out for 5 min at 75°C. Fmoc‐His(Trt)‐OH was coupled in lower temperatures (10 min, 50°C) to prevent racemization, whereas two coupling procedures were required for the incorporation Fmoc‐Arg (Pbf)‐OH (30 min, 50°C). The triazole incorporation was achieved by the diazo‐transfer reaction, followed by the CuAAC click reaction. For the diazo‐transfer, ISA·HCl (7 equiv.) was used as a 1.2 M solution in water. DIPEA (14 equiv.) prepared as a 3·10^−3^ M solution in DMF was used. CuSO_4_ was used as a catalyst (0.02 equiv.), and the reaction was carried out for 30 min at 80°C. For the CuAAC click reaction, [Cu(CH_3_CN)_4_]PF_6_ (0.5 equiv.) was used as a copper source and TBTA (0.5 equiv.) as a stabilizing agent both as 0.01 M solutions in DMF. The corresponding amino alkyne (5 equiv.) was dissolved in DMF (0.1 M) and added to the reaction as was DIPEA (1 equiv.). The CuAAC reaction was run for 1 h at 80°C, and the resin was washed with a 0.5% solution of diethyldithiocarbamate (6 × 4.5 ml) followed by DMF (4 × 4.5 ml), DCM (4 × 4.5 ml), and DMF (4 × 4.5 ml). After the completion of the sequence, a washing step of the resin was carried out with DCM (3 × 3 ml). The side chain protecting groups used were Boc (Trp), ^*t*^Bu (Tyr, Glu), Pbf (Arg), and Trt (Gln, His). The deprotection of the peptides and cleavage from the resin was performed using TFA/H_2_O/TIPS (95.0:2.5:2.5) for 2 h at RT on a shaker. The cleaved peptides were dried under a stream of argon, precipitated with ice‐cold MTBE (3 ml), and centrifuged. After two additional washes with ice‐cold MTBE followed by vortexing and sonication, and centrifugation, the crude product was isolated and analyzed by HPLC‐MS.

### 3.2 General notes

#### 3.2.1 Amino alkynes

**FIGURE 3:**
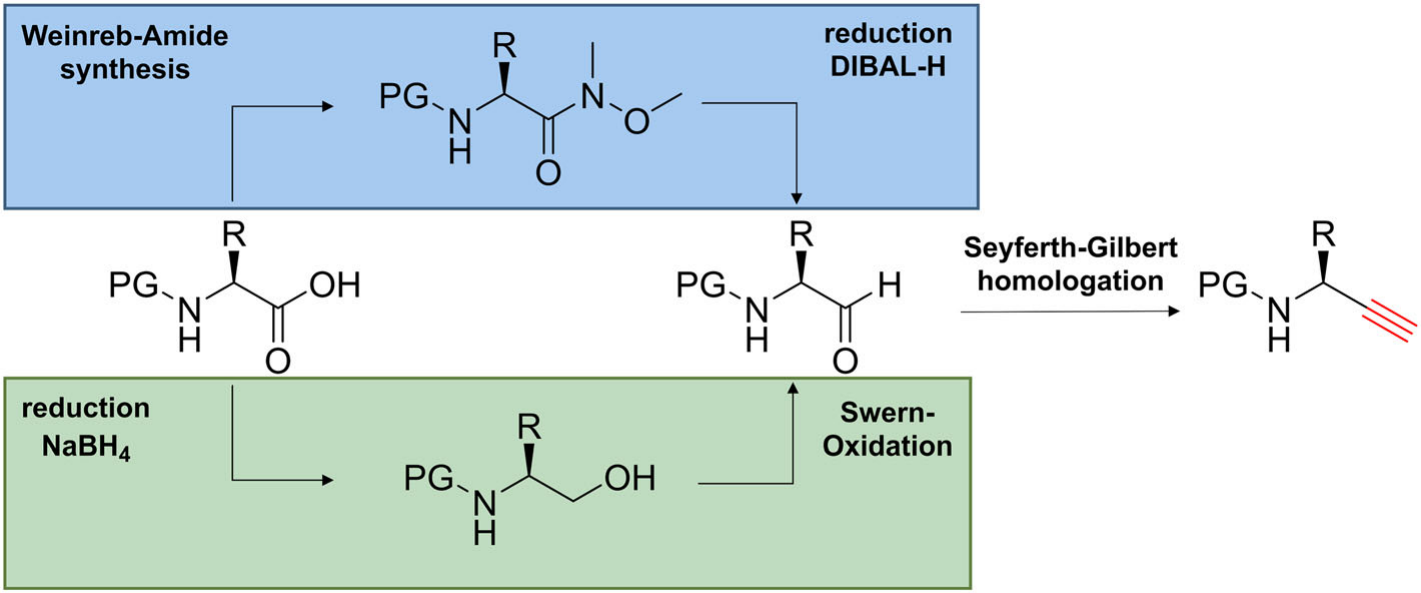
Representation of the two general strategies to synthesize α‐amino alkynes starting from amino acids.

The amino alkynes were synthesized from the corresponding amino acids following a three‐step synthetic route. These three steps include the formation of the Weinreb amide, the reduction to the corresponding amino aldehyde, and a Seyferth‐Gilbert homologation using the Bestmann‐Ohira reagent (Figure 3, blue route). In case of amino acids containing acidic side chain functionalities, an alternative route is recommended (Figure 3, green route). In this case, the carboxylic acid is reduced to the alcohol followed by Swern oxidation. Once the aldehyde is obtained, the homologation is performed with the Bestmann‐Ohira reagent as described above. If deprotection of the Fmoc protecting group is observed as a result of the presence of base (K_2_CO_3_) required for the Seyferth‐Gilbert homologation in the last step, the amino group of the obtained crude product can be protected again using Fmoc‐ONSu. These synthesis protocols can, at least in our hands, result in the partial racemization of the resulting amino alkynes (likely at the stage of the aldehyde intermediate).^26^ The presence of enantiomers is best analyzed by chiral‐HPLC.^27^ Alternatively, the amino alkynes can be coupled with an enantiomerically pure amino acid, followed by analysis of the diastereomeric purity of the resulting dipeptide by NMR or HPLC.^28^ The incorporation of the partially racemized amino alkynes in the peptide backbone via the CuAAC reaction results in the formation of a mixture of diastereomers. The desired product can be readily identified by the comparison of the diastereomeric ratio of the peptide (determined by UV‐HPLC) and the enantiomeric ration of the chiral amino alkyne.^25^ Alternatively, the (partially) racemized amino alkyne can be subjected to (semi‐)preparative chiral‐HPLC purification prior to use. All diasteromeric mixtures of final triazolo‐peptidomimetics studied by us so far could be separated efficiently by HPLC using a C18 column.

## Supporting information


**Figure S1:** Analysis of the stability of ISA·HCl in a) Millipore water, b) DMF, and c) DMSO.
**Figure S2:** Efficacy of Cu(I) + TBTA solution at 0 and 24 hours post preparation.
**Figure S3**: Structure and HPLC–MS analysis of H‐Gln^1^‐Trp^2^‐Ala^3^‐Val^4^‐Gly^5^ ***Ψ*[Trz]**His^6^‐Leu^7^‐Nle^8^‐NH_2._
**Figure S4**: Structure and HPLC–MS analysis of H‐Gln^1^‐Trp^2^‐Ala^3^‐Val^4^‐Gly^5^ ***Ψ*[Trz]**His^6^‐Leu^7^‐Nle^8^‐OH.
**Figure S5**: Structure and HPLC‐MS analysis of H‐Tyr^1^‐Gly^2^‐Gly^3^‐Phe^4^ ***Ψ*[Trz]**Leu^5^‐OH.
**Figure S6**: Structure and HPLC‐MS analysis of H‐Arg^1^ ***Ψ*[Trz]**Arg^2^‐Pro^3^‐Tyr^4^‐Ile^5^‐Leu^6^‐NH_2_
H‐Glu^1^ ***Ψ*[Trz]**Ala^2^‐Tyr^3^ ***Ψ*[Trz]**Gly^4^‐Trp^5^‐Nle^6^‐Gln^7^‐Phe^8^‐NH_2._
**Figure S7**: Structure and HPLC‐MS analysis of H‐Glu^1^ ***Ψ*[Trz]**Ala^2^‐Tyr^3^ ***Ψ*[Trz]**Gly^4^‐Trp^5^‐Nle^6^‐Gln^7^‐Phe^8^‐NH_2_.

**Data S1.** Supporting Information
