# Supplementary material for: Automated solid‐phase synthesis of metabolically stabilized triazolo‐peptidomimetics
Source: J Pept Sci. 2023 Mar 29;29(9):e3488. doi: 10.1002/psc.3488 (PMC10909554; doi:10.1002/psc.3488)
Supplement: Supplementary file 1 — Figure S1: Analysis of the stability of ISA·HCl in a) Millipore water, b) DMF, and c) DMSO. Figure S2: Efficacy of Cu(I) + TBTA solution at 0 and 24 hours post preparation. Figure S3: Structure and HPLC–MS analysis of H‐Gln1‐Trp2‐Ala3‐Val4‐Gly5 Ψ[Trz]His6‐Leu7‐Nle8‐NH2. Figure S4: Structure and HPLC–MS analysis of H‐Gln1‐Trp2‐Ala3‐Val4‐Gly5 Ψ[Trz]His6‐Leu7‐Nle8‐OH. Figure S5: Structure and HPLC‐MS analysis of H‐Tyr1‐Gly2‐Gly3‐Phe4 Ψ[Trz]Leu5‐OH. Figure S6: Structure and HPLC‐MS analysis of H‐Arg1 Ψ[Trz]Arg2‐Pro3‐Tyr4‐Ile5‐Leu6‐NH2 H‐Glu1 Ψ[Trz]Ala2‐Tyr3 Ψ[Trz]Gly4‐Trp5‐Nle6‐Gln7‐Phe8‐NH2. Figure S7: Structure and HPLC‐MS analysis of H‐Glu1 Ψ[Trz]Ala2‐Tyr3 Ψ[Trz]Gly4‐Trp5‐Nle6‐Gln7‐Phe8‐NH2. [file PSC-29-e3488-s002.docx]

**Automated Solid Phase Synthesis of Metabolically Stabilized Triazolo-Peptidomimetics**

**X. Guarrochena, ^1,2,3,4^ B. Kaudela, ^1,2,3^ T. L. Mindt ^1,3,4*^**

**Correspondence to: Thomas Mindt, Bioinorganic Radiochemistry, Institute of Inorganic Chemistry Faculty of Chemistry, University of Vienna, Josef-Holaubek-Platz 2 (UZAII), 1090 Vienna, Austria. Email:* [*thomas.mindt@univie.ac.at*](javascript:linkTo_UnCryptMailto('ocknvq,vjqocu0okpfvBwpkxkg0ce0cv');)

*^1^ Department of Inorganic Chemistry, Faculty of Chemistry, University of Vienna, Währinger Strasse 42, 1090 Vienna, Austria*

*^2^ Vienna Doctoral School in Chemistry, University of Vienna, Währinger Strasse 42, 1090 Vienna, Austria*

*^3^ Ludwig Boltzmann Institute Applied Diagnostics, AKH Wien c/o Sekretariat Nuklearmedizin,Waehringer Guertel 18-20, 1090 Vienna , Austria ^4^Department of Biomedical Imaging and Image Guided Therapy, Division of Nuclear Medicine, Medical University of Vienna, Waehringer Guertel 18-20, 1090 Vienna, Austria*

**1. Table of Content**

1. Table of content 1
2. Materials and reagents 2
3. Stability studies of ISA·HCl 3
4. Efficiency of Cu(I)+TBTA solution over time 4
5. HPLC-MS analysis of triazolo-peptidomimetics 5
6. References 10

**2. Materials and reagents**

Unless specified reagent grade chemicals were used. The L-configuration amino acids used in the peptide synthesis (Fmoc-Ala-OH, Fmoc-Cys(Trt), Fmoc-Gln(Trt)-OH, Fmoc-Glu(^t^Bu)-OH, Fmoc-Gly-OH, Fmoc-His(Trt)-OH, Fmoc-Ile-OH, Fmoc-Leu-OH, Fmoc-Lys(Boc)-OH, Fmoc-Nle-OH, Fmoc-Phe-OH, Fmoc-Thr(^t^Bu)-OH, Fmoc-Tyr-OH and Fmoc-Val-OH) were supplied by Iris Biotech as well as the Wang resin (loading 0.99 mmol/g). The The rink amide MBHA resin (loading 0.65 mmol/g) was purchased from Novabiochem. The diazotransfer reagent ISA·HCl was supplied by Fluorochem, the [Cu(CH_3_CN)_4_]PF_6_ catalyst by Sigma aldrich and the TBTA stabilizer by TCI. The amino alkynes used in the CuAAC reaction were synthesized as previously reported^1–3^. Reagent grade DMF (99.8%) and DCM (99.8%) were used is solid phase peptide synthesis. The stability studies of ISA·HCl were conducted in Milipore water, HPLC grade DMF (99.9%) and HPLC grade DMSO (99.9%). The peptides were synthesized with the Biotage™ Initiator+Alstra peptide synthesizer with microwave function. The crude purity of the peptides was determined by HPLC-MS using the Agilent 1260 Infinity II system equipped with an automatic sampler (1260 Vialsampler), the 1260 VWD UV-detector and the LC/MSC mass-spectrometer. The analysis was performed using the ACQUITY UPLC^®^ Peptide BEH C18 (300 Å, 1.7 um, 2.1 mm x 100mm) reverse phase column. The stability of ISA·HCl was assessed using the ACQUITY UPLC^®^ BEH C18 Column (300 Å, 1.7 um, 3 mm x 50mm).

**3. Stability studies of ISA·HCl**

ISA·HCl was dissolved in either Millipore water, DMF (HPLC grade) or DMSO (HPLC grade) at a concentration of 1.2M. Samples were taken at 0,1,2,4, and 24 hours post preparation and were analysed by HPLC-MS. The Acquity UHPLC BEH C18 column (300 Å, 1.7 um, 3.0 x 50 mm) was used for the stability assay with a gradient of 5-95%, 0.1% FA in ACN over 7 minutes with a flow rate of 0.6 mL/min.

**A) Milipore water**

**
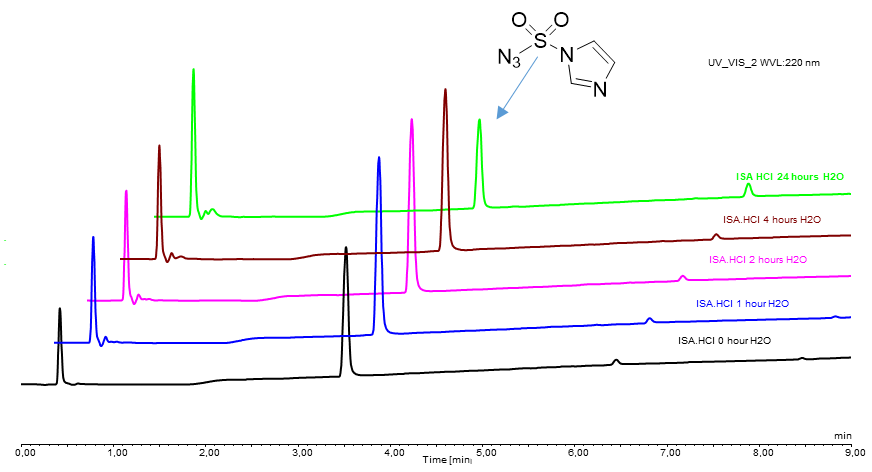
**

**B) DMF**

**
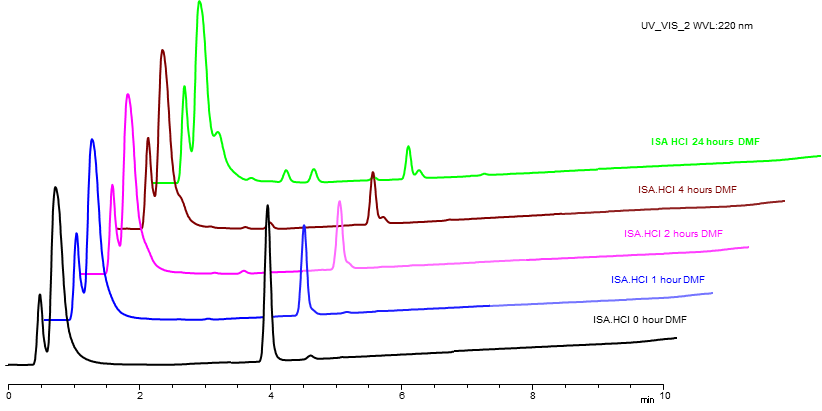
**

**C) DMSO**

**
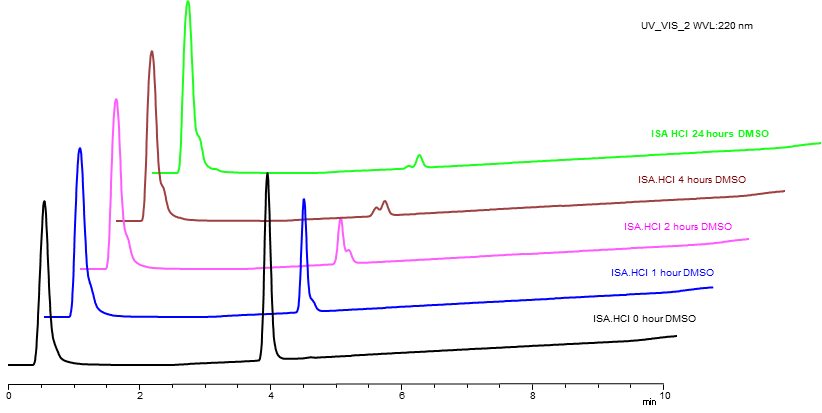
**

**Figure S1:** Analysis of the stability of ISA·HCl in a) Millipore water, b) DMF, and c) DMSO

**4. Efficiency of Cu(I)+TBTA solution over time**

The Cu(I)+TBTA solution (0.01 M) was prepared in DMF and was used immediately and 24 hours after preparation in the synthesis of H-Gly*Ψ*[Tz]His-Leu-Nle-NH_2_ starting from N_3_-His-Leu-Nle-NH_2_.

0,20

0,40

0,60

0,80

1,00

1,20

1,40

1,60

1,80

2,00

2,20

2,40

2,60

2,80

3,00

3,20

3,40

3,60

3,80

4,00

4,20

4,40

4,60

4,80

5,00

min

0 hours

24 hours

H-Gly*Ψ*[Tz]His-Leu-Nle-NH_2_

**Figure S2:** Efficacy of Cu(I)+TBTA solution at 0 and 24 hours post preparation.

**5. LC-MS analysis of triazolo-peptidomimetics**

H-Gln^1^-Trp^2^-Ala^3^-Val^4^-Gly^5^***Ψ*[Trz]**His^6^-Leu^7^-Nle^8^-NH_2_

Gradient: 10-70%, 0.1% FA in ACN over 13 minutes with a flow rate of 0.2 mL/min.

MS *m/z* [M+H]^+^ and [M+2H]^+2^ calculated for C_45_H_67_N_15_O_8_: 946.5 and 473.7, respectively. Found 946.8 and 474.0


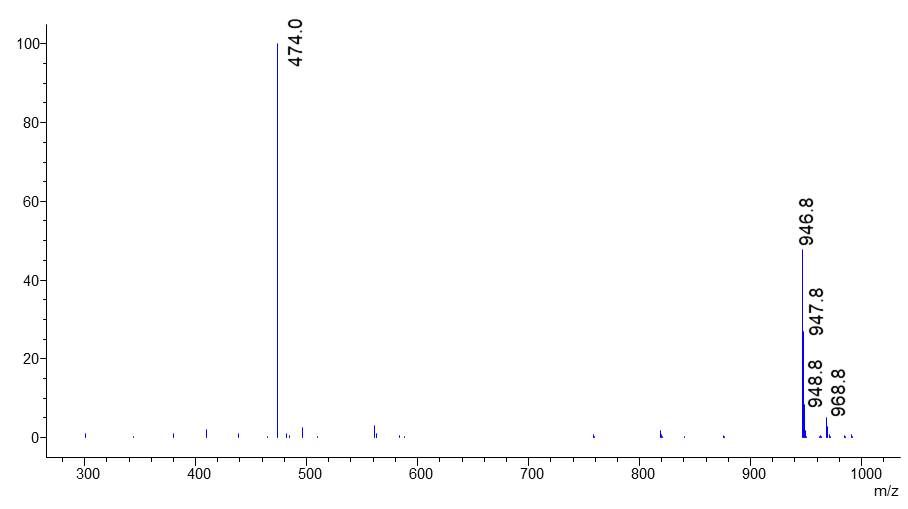


min

2

4

6

8

10

12

14

16

mAU

0

200

400

600

800

1000

1200

1400

8.61

8.94

9.08

9.48

10.34

11.11

Wavelength = 220 nm

**Figure S3**: Structure and HPLC-MS analysis of H-Gln^1^-Trp^2^-Ala^3^-Val^4^-Gly^5^***Ψ*[Trz]**His^6^-Leu^7^-Nle^8^-NH_2_

H-Gln^1^-Trp^2^-Ala^3^-Val^4^-Gly^5^***Ψ*[Trz]**His^6^-Leu^7^-Nle^8^-OH

Gradient: 10-70%, 0.1% FA in ACN over 13 minutes with a flow rate of 0.2 mL/min.

MS *m/z* [M+H]^+^ and [M+2H]^+2^ calculated for C_45_H_66_N_14_O_9_^+^, 947.5 and 474.26, respectively. Found 947.8 and 474.5


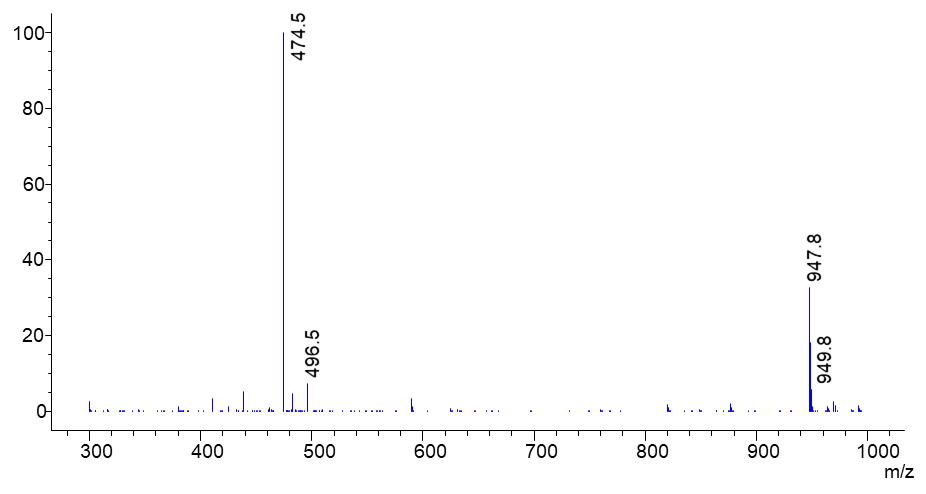


min

2

4

6

8

10

12

14

16

mAU

0

200

400

600

800

8.74

8.97

9.11

9.70

Wavelength = 220 nm

**Figure S4**: Structure and HPLC-MS analysis of H-Gln^1^-Trp^2^-Ala^3^-Val^4^-Gly^5^***Ψ*[Trz]**His^6^-Leu^7^-Nle^8^-OH

H-Tyr^1^-Gly^2^-Gly^3^-Phe^4^***Ψ*[Trz]**Leu^5^-OH

Gradient: 10-60%, 0.1% FA in ACN over 10 minutes with a flow rate of 0.2 mL/min.

MS *m/z* [M+H]^+^ and [M+2H]^+2^ calculated for C_29_H_37_N_7_O_6_^+^, 580.3 and 290.1, respectively. Found 580.4 and 290.8


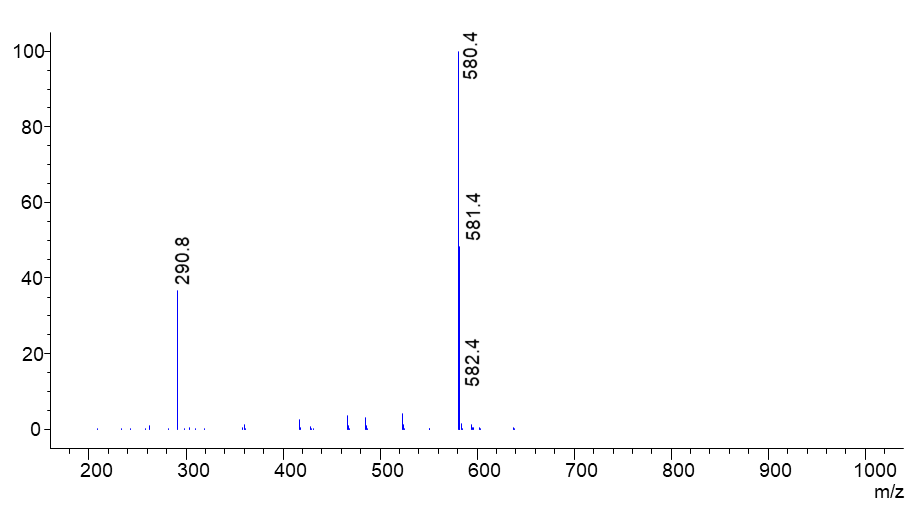


min

2

4

6

8

10

12

mAU

0

200

400

600

800

1000

1200

1400

6.93

7.14

8.34

8.48

8.92

9.13

Wavelength = 220 nm

**Figure S5**: Structure and HPLC-MS analysis of H-Tyr^1^-Gly^2^-Gly^3^-Phe^4^***Ψ*[Trz]**Leu^5^-OH

H-Arg^1^***Ψ*[Trz]**Arg^2^-Pro^3^-Tyr^4^-Ile^5^-Leu^6^-NH_2_

Gradient: 5-50%, 0.1% FA in ACN over 13 minutes with a flow rate of 0.2 mL/min.

MS *m/z* [M+H]^+^, [M+2H]^+2^ and [M+3H]^+3^ calculated for C_39_H_65_N_15_O_6_, 840.5, 420.8 and 280.8, respectively. Found 840.7, 420.9 and 281.0

min

2

4

6

8

10

12

14

16

18

mAU

0

100

200

300

400

500

600

700

800

8.67

9.91

10.94

11.69

12.91

m/z

200

300

400

500

600

700

800

900

1000

0

20

40

60

80

100

841.7

275.4

355.9

840.7

281.0

420.9

**Figure S6**: Structure and HPLC-MS analysis of H-Arg^1^***Ψ*[Trz]**Arg^2^-Pro^3^-Tyr^4^-Ile^5^-Leu^6^-NH_2_

H-Glu^1^***Ψ*[Trz]**Ala^2^-Tyr^3^***Ψ*[Trz]**Gly^4^-Trp^5^-Nle^6^-Gln^7^-Phe^8^-NH_2_

Gradient: 20-65%, 0.1% FA in ACN over 13 minutes with a flow rate of 0.2 mL/min.

MS *m/z* [M+H]^+^ and [M+2H]^+2^ calculated for C_51_H_62_N_14_O_11_, 1047.5 and 524.2, respectively. Found 1047.8 and 524.5.

min

2

4

6

8

10

12

14

16

mAU

0

100

200

300

400

500

600

8.58

8.92

10.07

10.27

Wavelength = 220 nm


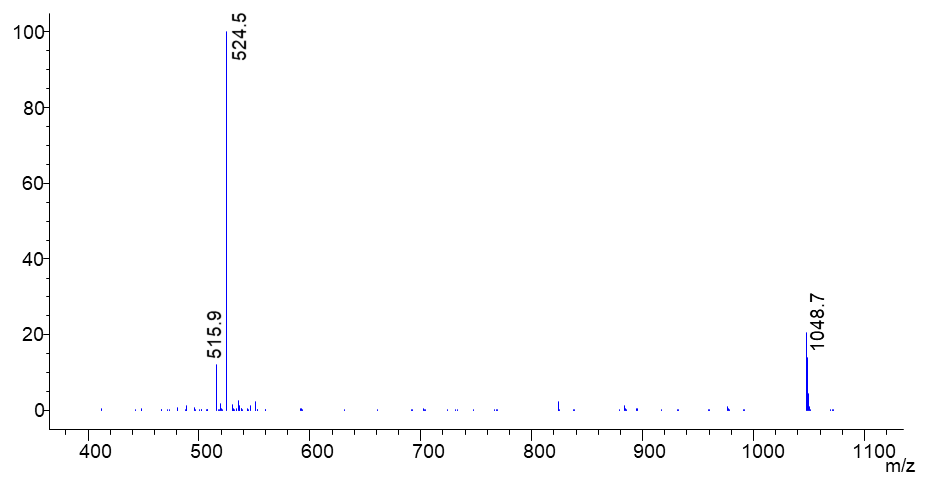


**Figure S7**: Structure and HPLC-MS analysis of H-Glu^1^***Ψ*[Trz]**Ala^2^-Tyr^3^***Ψ*[Trz]**Gly^4^-Trp^5^-Nle^6^-Gln^7^-Phe^8^-NH_2_

**5. References**

1. Grob NM, Häussinger D, Deupi X, Schibli R, Behe M, Mindt TL. Triazolo-Peptidomimetics: Novel Radiolabeled Minigastrin Analogs for Improved Tumor Targeting. *J. Med. Chem.* **2020**;63:4484–4495.

2. Valverde IE, Vomstein S, Fischer CA, Mascarin A, Mindt TL. Probing the Backbone Function of Tumor Targeting Peptides by an Amide-to-Triazole Substitution Strategy. *J. Med. Chem.* **2015**;58:7475–7484.

3. Valverde IE, Bauman A, Kluba CA, Vomstein S, Walter MA, Mindt TL. 1,2,3-Triazoles as Amide Bond Mimics: Triazole Scan Yields Protease-Resistant Peptidomimetics for Tumor Targeting. *Angew Chemie - Int. Ed.* **2013**;52:8957–8960.
