# Supplementary material for: Automated solid‐phase synthesis of metabolically stabilized triazolo‐peptidomimetics
Source: J Pept Sci. 2023 Mar 29;29(9):e3488. doi: 10.1002/psc.3488 (PMC10909554; doi:10.1002/psc.3488)
Supplement: Supplementary file 2 — Data S1. Supporting Information [file PSC-29-e3488-s001.pdf]

Initiator+ Alstra Peptide Sequence Summary

User:
Date: 2021-11-04 11:09
Vial: 10 mL
Resin: Rink amide ChemMatrix
Resin Functionality: Amide
Loading: 0.65 mmol/g
Resin Functionality Molecular Weight: 17.0 g/mol
Molecular Weight: 919.5 g/mol
Product Weight: 0.028 g
Quantity: 0.046 g
Scale: 0.03 mmol
Notes:
Sequence:

Q W A V G_{*} H L L_{Nle} # 1

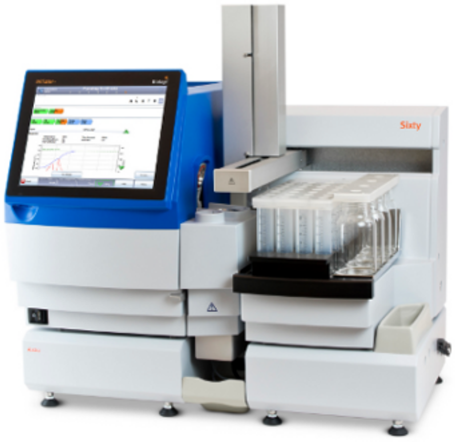

Table of Contents

Calculation Table	3
1 **L$_{Nle}$** (Fmoc-Norleucine-OH)	4
2 **L** (Fmoc-Leu-OH)	5
3 **H** (Fmoc-His(Trt)-OH)	6
4 **G*** (Fmoc-Gly-Alk)	7
5 **V** (Fmoc-Val-OH)	8
6 **A** (Fmoc-Ala-OH)	9
7 **W** (Fmoc-Trp(Boc)-OH)	10
8 **Q** (Fmoc-Gln(Trt)-OH)	11

Calculation Table

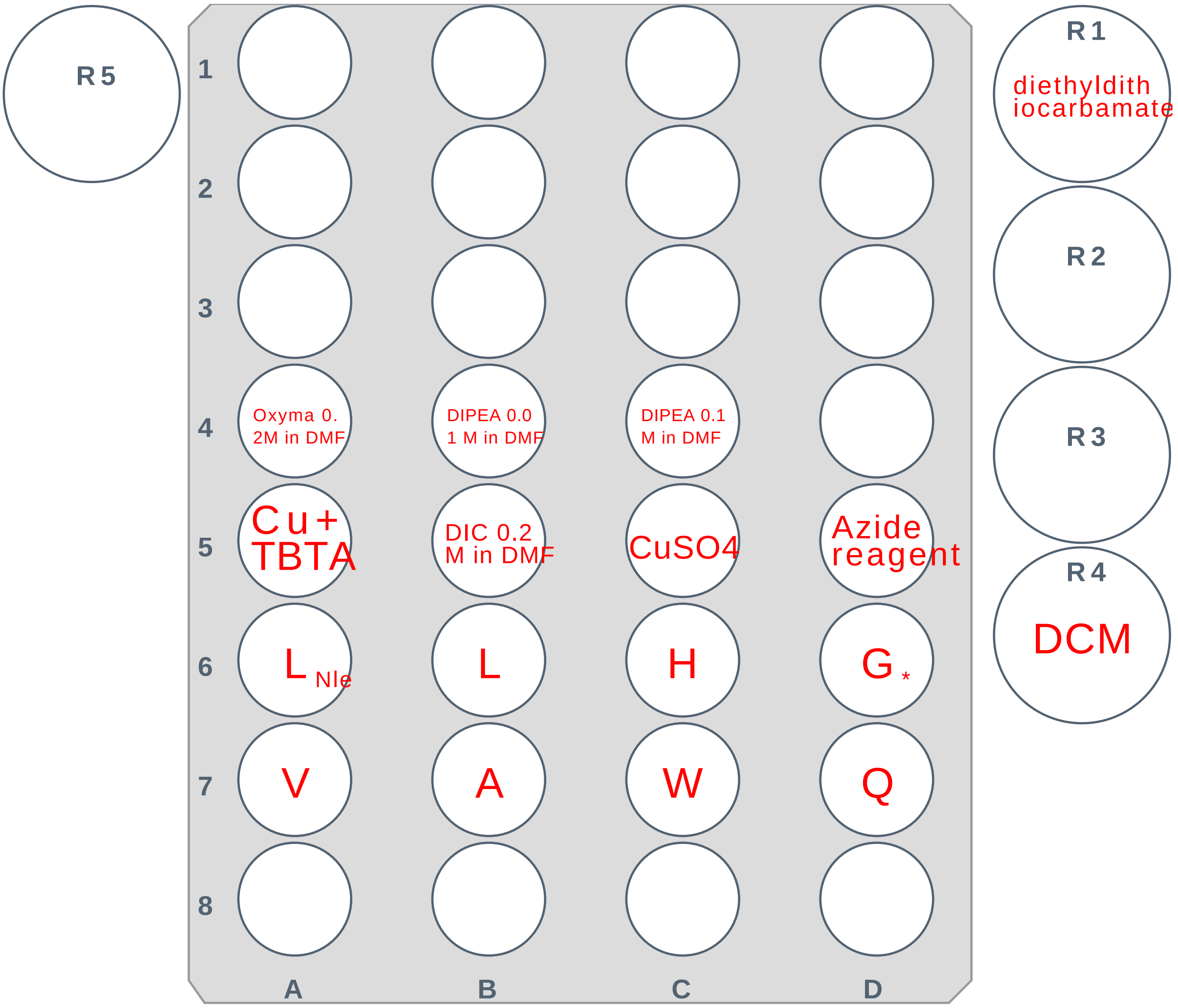

Pos	Acid	Chemical Name	Equivalents	Mol Mass [g/mol]	Mass [g]	Volume [mL]	Dissolve Volume [mL]	Concentration [mol/L]	Total Volume [mL]
A:4		Oxyma 0.2M in DMF	5.0	142.1	0.152		5.35	0.2	5.35
A:5		Cu+TBTA 0.01M in DMF	0.5	0.0			1.6	0.01	1.6
A:6	L_{Nle}	Fmoc-Norleucine-OH	5.0	353.4	0.057		1.555	0.1	1.6
A:7	V	Fmoc-Val-OH	5.0	339.4	0.054		1.557	0.1	1.6
B:4		DIPEA 0.01 M in DMF	1.0	129.2		0.005	3.095	0.01	3.1

Pos	Acid	Chemical Name	Equivalents	Mol Mass [g/mol]	Mass [g]	Volume [mL]	Dissolve Volume [mL]	Concentration [mol/L]	Total Volume [mL]
B:5		DIC 0.2M in DMF	5.0	126.2		0.166	5.184	0.2	5.35
B:6	L	Fmoc-Leu-OH	5.0	353.4	0.057		1.555	0.1	1.6
B:7	A	Fmoc-Ala-OH	5.0	311.3	0.05		1.561	0.1	1.6
C:4		DIPEA 0.1 M in DMF	14.0	129.3		0.075	4.225	0.1	4.3
C:5		CuSO4 in DMF			0.0				1.1
C:6	H	Fmoc-His(Trt)-OH	5.0	619.7	0.099		1.518	0.1	1.6
C:7	W	Fmoc-Trp(Boc)-OH	5.0	526.6	0.084		1.531	0.1	1.6
D:5		Imidazolyl-1-sulfonyl hydrochloride 1.2 M in water	7.0	209.6	0.069		0.275	1.2	0.275
D:6	G*	Fmoc-Gly-Alk	5.0	277.3	0.044		1.565	0.1	1.6
D:7	Q	Fmoc-Gln(Trt)-OH	5.0	610.7	0.098		1.519	0.1	1.6
R1		0.5% carbamate in DMF		225.3	0.0		32.0	0.0	32.0
R4		DCM				0.0			50.0
S1		DMF				0.0			513.9
S2		DMF				0.0			86.0
S3		20% Piperidine in DMF	300.0	85.2		34.139	138.661	2.0	172.8

Cycle No: 1

L$_{Nle}$ (Fmoc-Norleucine-OH)

Swelling DMF, 2021-11-04 11:09

Reaction: Temp: 70°C **Time (mm:ss):** 20:00 **Oscillating Mixer:** On

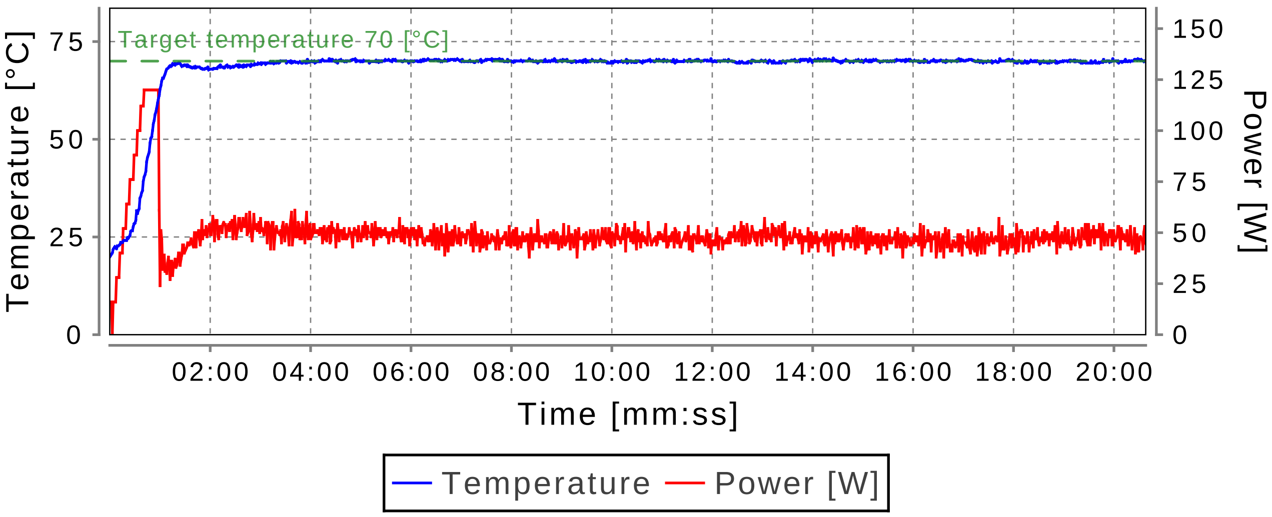

20% Piperidine , 2021-11-04 11:32

Reaction: Temp: Room Temperature **Time (mm:ss):** 03:00 **Oscillating Mixer:** On **Interval Mixing On/Off:** 10/15

Reaction: Temp: Room Temperature **Time (mm:ss):** 10:00 **Oscillating Mixer:** On **Interval Mixing On/Off:** 10/15

DIC/Oxyma, 2021-11-04 11:57

Reaction: Temp: 75°C **Time (mm:ss):** 05:00 **Oscillating Mixer:** On

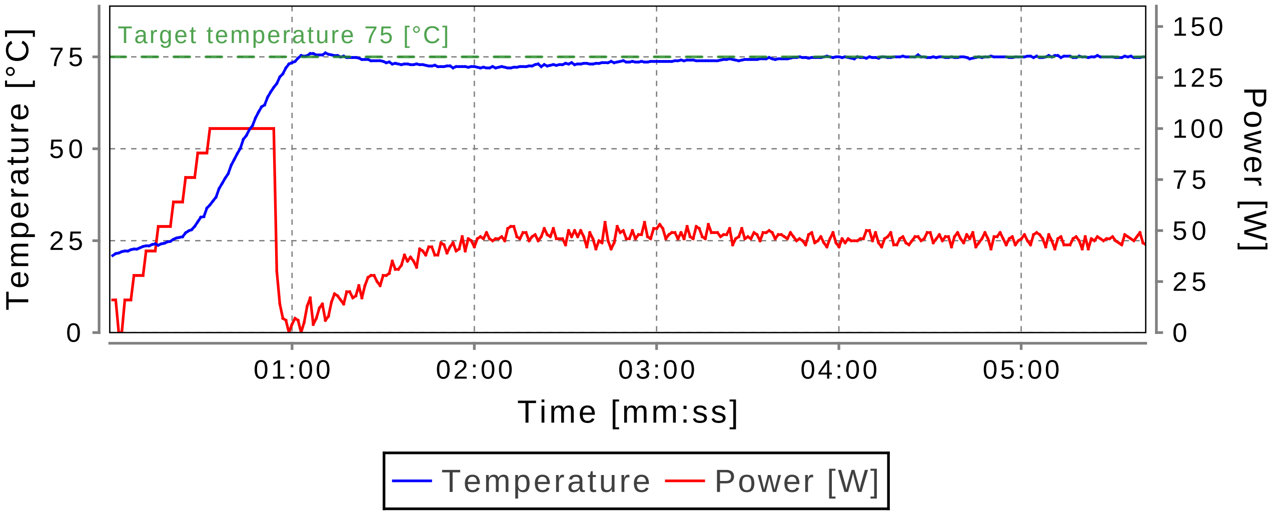

20% Piperidine , 2021-11-04 12:14

Reaction: Temp: Room Temperature **Time (mm:ss):** 03:00 **Oscillating Mixer:** On **Interval Mixing On/Off:** 10/15

Reaction: Temp: Room Temperature **Time (mm:ss):** 10:00 **Oscillating Mixer:** On **Interval Mixing On/Off:** 10/15

Cycle No: 2

L (Fmoc-Leu-OH)

DIC/Oxyma, 2021-11-04 12:40

Reaction: Temp: 75°C **Time (mm:ss):** 05:00 **Oscillating Mixer:** On

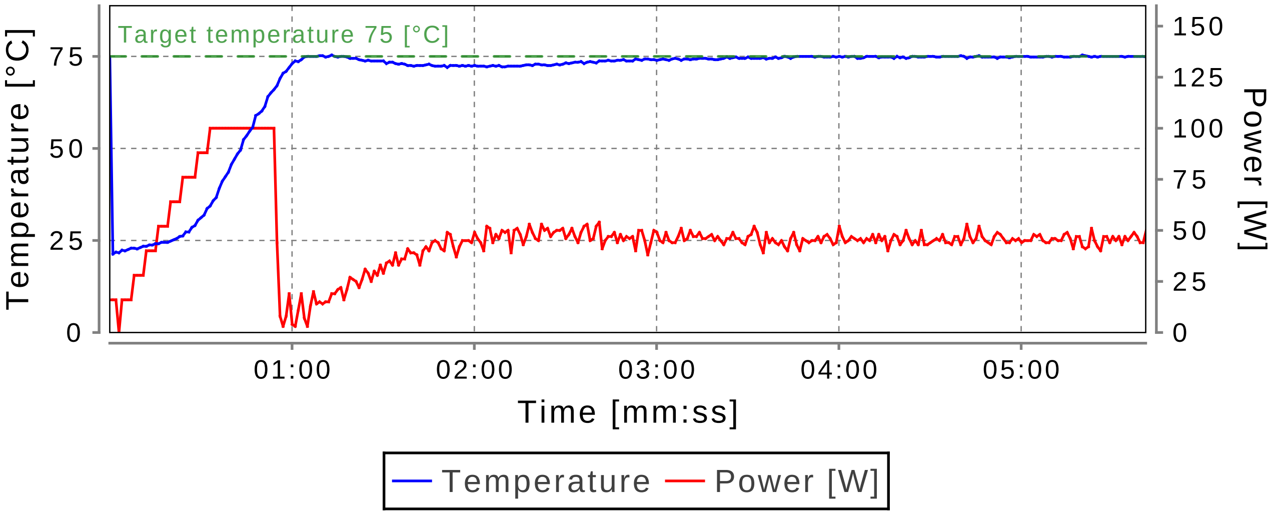

20% Piperidine , 2021-11-04 12:57

Reaction: Temp: Room Temperature **Time (mm:ss):** 03:00 **Oscillating Mixer:** On **Interval Mixing On/Off:** 10/15

Reaction: Temp: Room Temperature **Time (mm:ss):** 10:00 **Oscillating Mixer:** On **Interval Mixing On/Off:** 10/15

Cycle No: 3

H (Fmoc-His(Trt)-OH)

DIC/Oxyma, 2021-11-04 13:22

Reaction: Temp: 75°C **Time (mm:ss):** 05:00 **Oscillating Mixer:** On

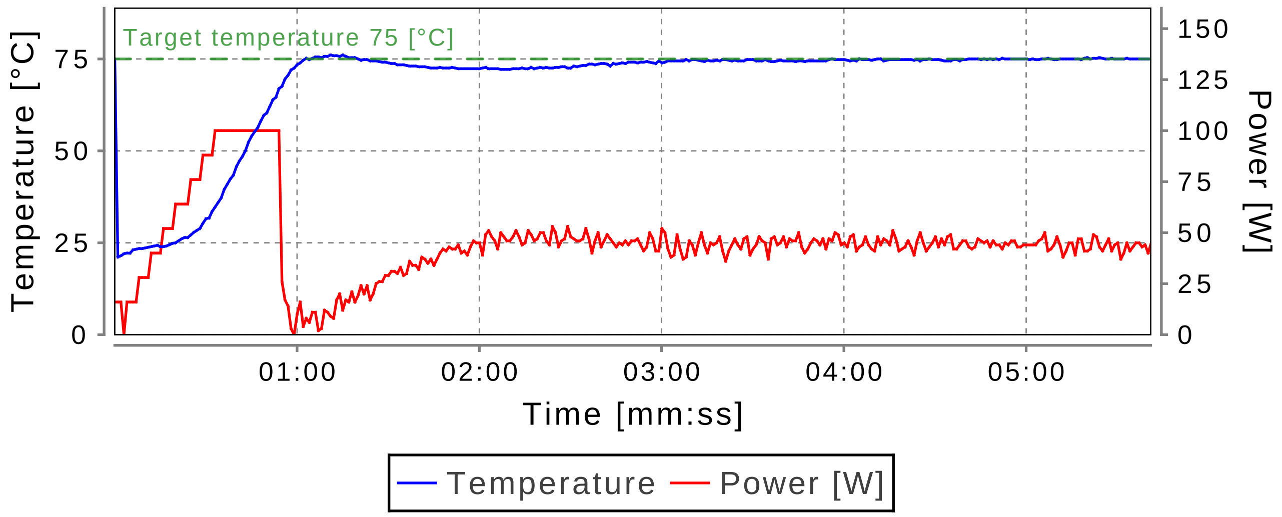

20% Piperidine , 2021-11-04 13:40

Reaction: Temp: Room Temperature **Time (mm:ss):** 03:00 **Oscillating Mixer:** On **Interval Mixing On/Off:** 10/15

Reaction: Temp: Room Temperature **Time (mm:ss):** 10:00 **Oscillating Mixer:** On **Interval Mixing On/Off:** 10/15

Diazotransfer 1.2M in water with CuSO4, 2021-11-04 14:05

Reaction: Temp: 80°C **Time (mm:ss):** 30:00 **Oscillating Mixer:** On

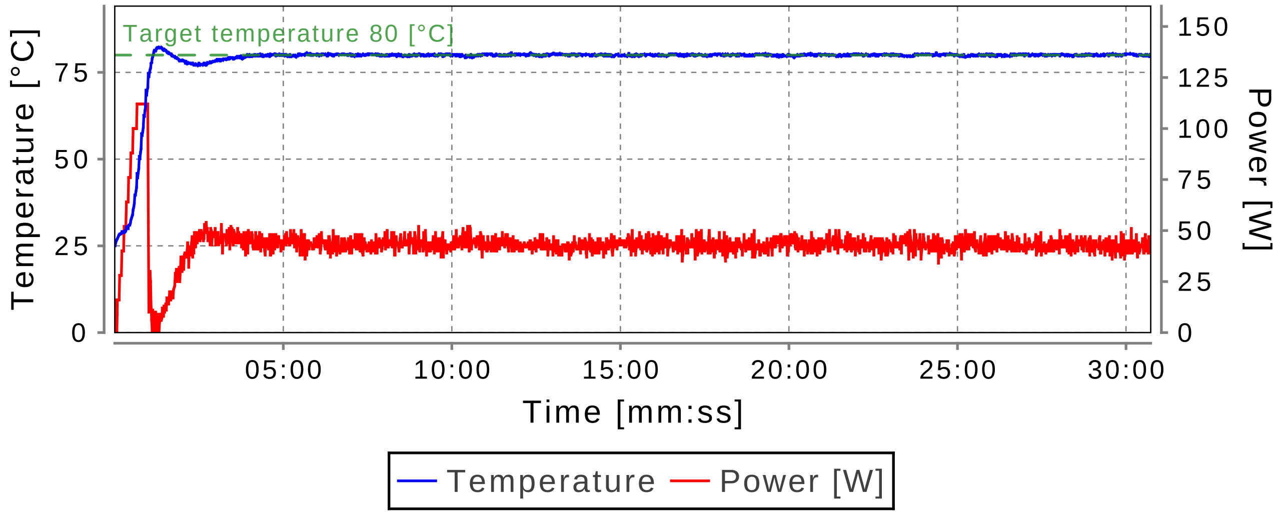

Cycle No: 4

G* (Fmoc-Gly-Alk)

CuAAC, 2021-11-04 14:49

Reaction: Temp: 70°C **Time (mm:ss):** 60:00 **Oscillating Mixer:** On

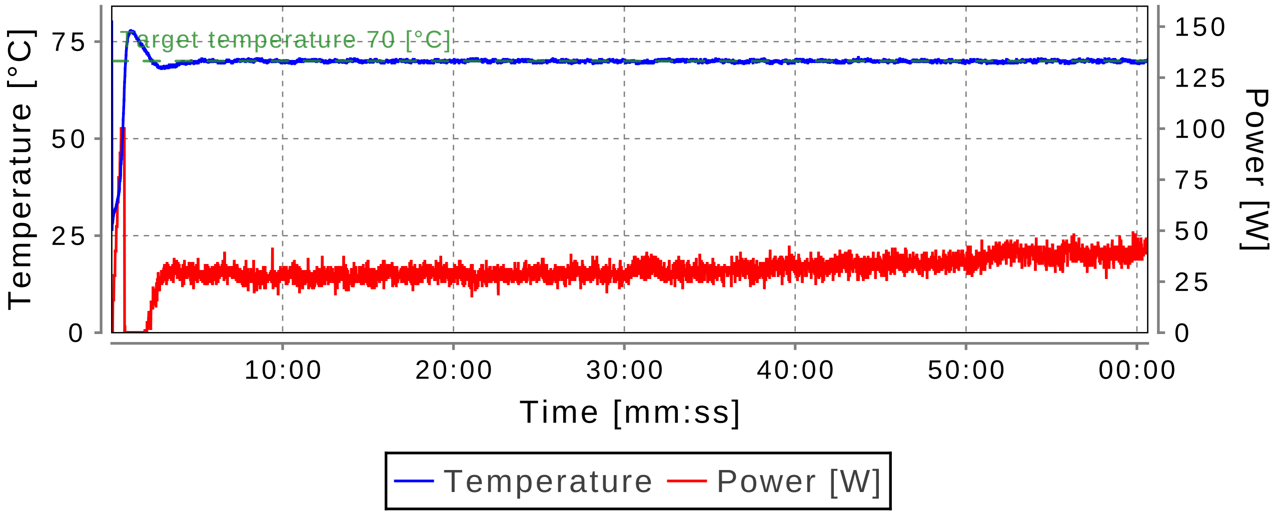

20% Piperidine , 2021-11-04 17:11

Reaction: Temp: Room Temperature **Time (mm:ss):** 03:00 **Oscillating Mixer:** On **Interval Mixing On/Off:** 10/15

Reaction: Temp: Room Temperature **Time (mm:ss):** 10:00 **Oscillating Mixer:** On **Interval Mixing On/Off:** 10/15

Cycle No: 5
V (Fmoc-Val-OH)
DIC/Oxyma, 2021-11-04 17:36
Reaction: **Temp:** 75°C **Time (mm:ss):** 05:00 **Oscillating Mixer:** On

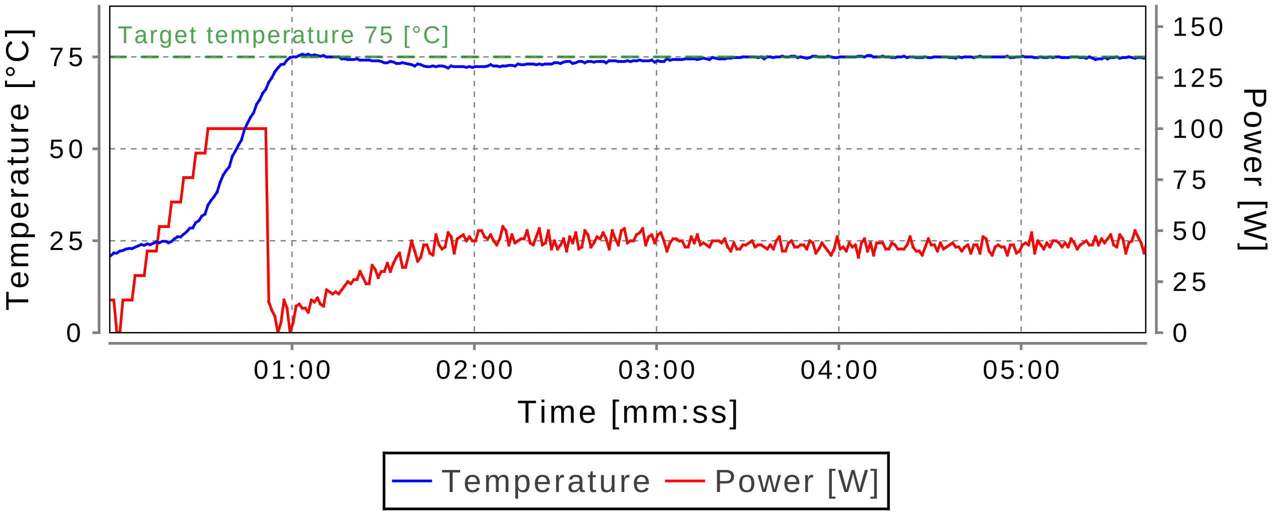

20% Piperidine , 2021-11-04 17:53
Reaction: **Temp:** Room Temperature **Time (mm:ss):** 03:00 **Oscillating Mixer:** On **Interval Mixing On/Off:** 10/15
Reaction: **Temp:** Room Temperature **Time (mm:ss):** 10:00 **Oscillating Mixer:** On **Interval Mixing On/Off:** 10/15

Cycle No: 6

A (Fmoc-Ala-OH)

DIC/Oxyma, 2021-11-04 18:18

Reaction: Temp: 75°C **Time (mm:ss):** 05:00 **Oscillating Mixer:** On

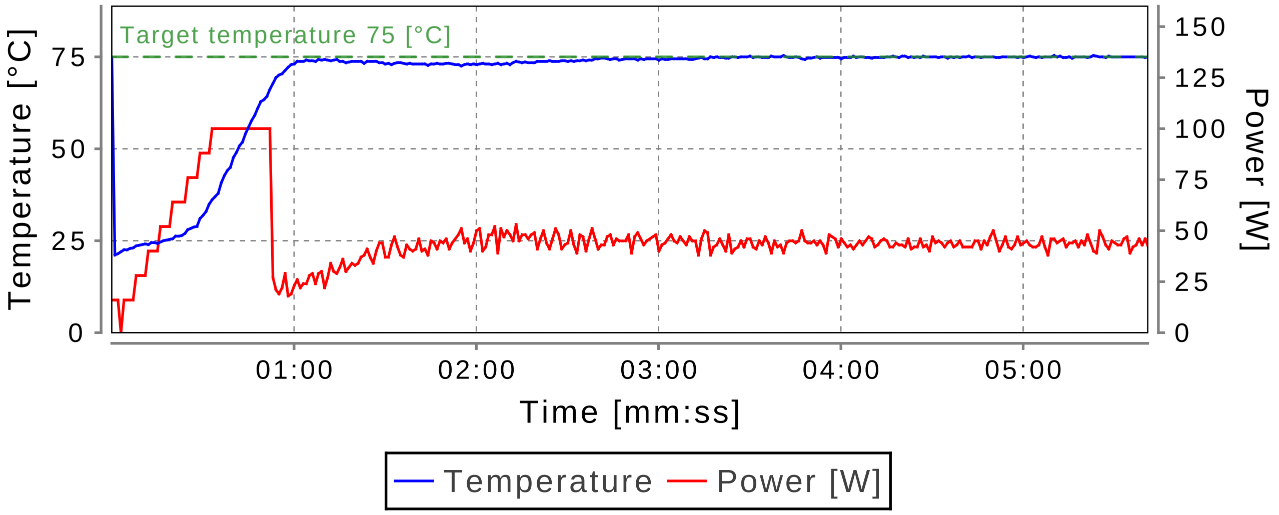

20% Piperidine , 2021-11-04 18:36

Reaction: Temp: Room Temperature **Time (mm:ss):** 03:00 **Oscillating Mixer:** On **Interval Mixing On/Off:** 10/15

Reaction: Temp: Room Temperature **Time (mm:ss):** 10:00 **Oscillating Mixer:** On **Interval Mixing On/Off:** 10/15

Cycle No: 7

W (Fmoc-Trp(Boc)-OH)

DIC/Oxyma, 2021-11-04 19:01

Reaction: **Temp:** 75°C **Time (mm:ss):** 05:00 **Oscillating Mixer:** On

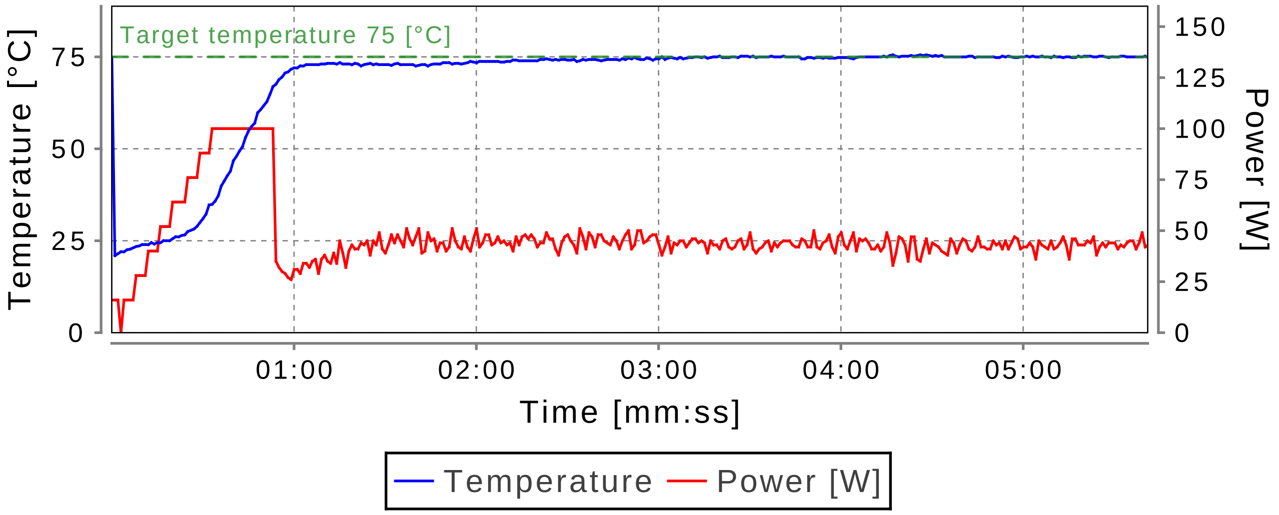

20% Piperidine , 2021-11-04 19:19

Reaction: **Temp:** Room Temperature **Time (mm:ss):** 03:00 **Oscillating Mixer:** On **Interval Mixing On/Off:** 10/15

Reaction: **Temp:** Room Temperature **Time (mm:ss):** 10:00 **Oscillating Mixer:** On **Interval Mixing On/Off:** 10/15

Cycle No: 8
Q (Fmoc-Gln(Trt)-OH)
DIC/Oxyma, 2021-11-04 19:44
Reaction: Temp: 75°C **Time (mm:ss):** 05:00 **Oscillating Mixer:** On

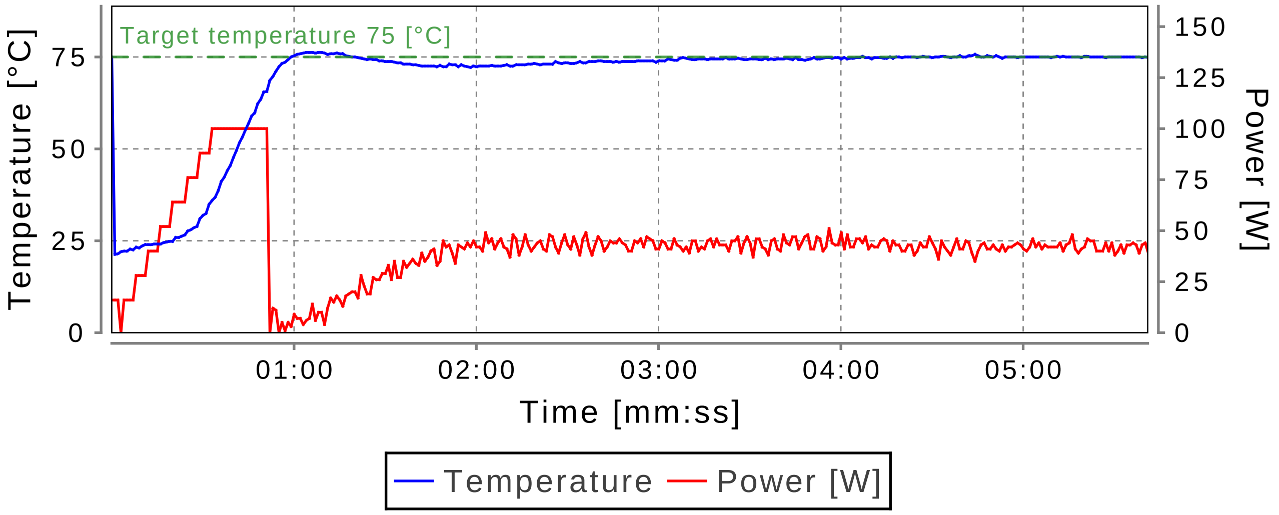

20% Piperidine , 2021-11-04 20:02
Reaction: Temp: Room Temperature **Time (mm:ss):** 03:00 **Oscillating Mixer:** On **Interval Mixing On/Off:** 10/15
Reaction: Temp: Room Temperature **Time (mm:ss):** 10:00 **Oscillating Mixer:** On **Interval Mixing On/Off:** 10/15
Pre-cleavage wash DCM , 2021-11-04 20:27
